# Development of a nomogram to predict acute liver injury in children with *Mycoplasma pneumoniae* pneumonia

**DOI:** 10.3389/fped.2026.1809544

**Published:** 2026-08-18

**Authors:** Shisi Xiong, Yanlin Tan, Junmei Bian, Xingxing Bao, Jiajun Zhou, Min Liang

**Affiliations:** Department of Pediatrics, Wuhan Third Hospital, Wuhan City, Hubei Province, China

**Keywords:** lasso-Logistic regression, multivariate logistic regression, retrospective cohort study, risk prediction, training set, validation set

## Abstract

**Objective:**

To construct and validate a nomogram for predicting acute liver injury in children with *Mycoplasma pneumoniae* pneumonia (MPP).

**Methods:**

This retrospective study included 964 hospitalized children with confirmed MPP from January 2021 to December 2024. Acute liver injury was defined as the study endpoint. Missing data were handled by multiple imputation (*m* = 5). The cohort was divided into a training set and a validation set at a ratio of 7:3 using stratified random sampling. Least absolute shrinkage and selection operator (LASSO)-logistic regression was used for feature selection, and the selected variables were entered into multivariable logistic regression to construct a nomogram. Model performance was assessed using receiver operating characteristic (ROC) curves, calibration curves, and decision curve analysis (DCA).

**Results:**

Among the 964 children, 131 developed acute liver injury, corresponding to an incidence of 13.6%. The cohort was divided into a training set of 676 children, including 92 with acute liver injury, and a validation set of 288 children, including 39 with acute liver injury. Baseline characteristics were comparable between the training and validation sets (*P* > 0.05). LASSO regression identified 11 candidate predictors: gender, age, body temperature ≥37.5 °C, history of allergy, neutrophil percentage, lymphocyte percentage, D-dimer, total protein, *γ*-glutamyl transpeptidase (GGT), lactate dehydrogenase (LDH), and creatine kinase isoenzyme MB (CK-MB). In multivariable analysis, female gender, GGT, LDH, and CK-MB were independently associated with an increased risk of liver injury, whereas age was negatively associated with liver injury. D-dimer showed marginal statistical significance. The nomogram showed good discrimination, with an area under the curve (AUC) of 0.837 in the training set and 0.874 in the validation set. Calibration curves showed acceptable agreement between predicted and observed risks, and DCA suggested potential clinical net benefit within a certain threshold range.

**Conclusion:**

This nomogram, based on routinely available clinical and laboratory indicators, may help identify children with MPP at increased risk of acute liver injury and support early liver function monitoring. Further prospective multicenter validation is needed.

## Introduction

*Mycoplasma pneumoniae* (MP) is an important cause of community-acquired pneumonia in children and may lead to severe or refractory disease and increased hospitalization burden (1, 2). In addition to respiratory involvement, MP infection can affect multiple extrapulmonary organs, including the hepatobiliary, cardiovascular, neurological, hematological, and vascular systems (3–5). Acute liver injury is a clinically relevant extrapulmonary manifestation of *Mycoplasma pneumoniae* pneumonia (MPP), but it may be overlooked at an early stage because affected children can be asymptomatic or present only with nonspecific manifestations (6–8).

Liver injury associated with MPP may present predominantly as aminotransferase elevation or, less commonly, as cholestatic abnormalities. Its pathogenesis is likely multifactorial and may involve immune-mediated injury, systemic inflammation, endothelial dysfunction, coagulation activation, hypoxia, microcirculatory disturbance, and medication exposure (5–7). Early identification of children at increased risk is therefore important for timely liver function monitoring and appropriate clinical management.

Previous studies have explored factors associated with liver injury in severe MPP and indicators related to extrapulmonary complications. Yu et al. (9) identified clinical and laboratory factors associated with liver injury in children with severe MPP. Zhang et al. (10) and Bao et al. (11) reported associations between inflammatory or tissue injury-related markers and severe clinical phenotypes, whereas Song et al. (12) evaluated inflammatory indices for predicting extrapulmonary complications. Prediction models based on machine learning or other complex approaches have also been developed for organ injury in severe MPP and infection-related liver injury (13, 14). However, most previous studies focused on severe MPP, overall extrapulmonary complications, or general organ damage rather than acute liver injury in the broader population of children with MPP. In addition, some existing models rely on complex variables or procedures that may limit their interpretability and bedside applicability.

Therefore, this study aimed to develop and internally validate a clinically interpretable nomogram for predicting acute liver injury in children with MPP using routinely available clinical and laboratory variables. Least absolute shrinkage and selection operator regression was used to select candidate predictors, followed by multivariable logistic regression to construct the model (15). Model performance was evaluated in terms of discrimination, calibration, and clinical net benefit in the training and validation sets.

## Methods

### Study subjects

This was a single-center retrospective cohort study. Children who were hospitalized and diagnosed with MPP at Wuhan Third Hospital from January 2021 to December 2024 were enrolled. The study outcome was the occurrence of acute liver injury (yes/no). The overall cohort was divided into a training set and a validation set at a ratio of 7:3 using stratified random sampling based on the study outcome. The training set was used for model development, and the validation set was used for internal validation. This study was reviewed and approved by the Medical Ethics Committee of Wuhan Third Hospital (approval No. KY2025-026; approval date: April 2, 2025). The study was conducted in accordance with the ethical principles of the Declaration of Helsinki.

### Diagnostic criteria for MPP

The diagnosis of MPP was based on compatible clinical manifestations, radiological evidence of pneumonia, and laboratory evidence of MP infection. Compatible clinical manifestations included fever, cough, or other respiratory symptoms. Radiological evidence was determined by chest imaging findings suggestive of pneumonia. Laboratory evidence of MP infection was defined as a positive serum MP-specific IgM antibody test and/or positive MP nucleic acid detection in respiratory tract specimens, according to the routine diagnostic procedures of the clinical laboratory of Wuhan Third Hospital. All microbiological tests were performed by trained laboratory personnel in accordance with the manufacturer's instructions and the standard operating procedures of the hospital laboratory.

### Collection of clinical data

Clinical and laboratory data were retrospectively extracted from the hospital electronic medical record system and laboratory information system. Prespecified candidate predictors included demographic characteristics (gender and age), clinical characteristics (body temperature ≥37.5 °C and history of allergy), routinely available hematological parameters [white blood cell count (WBC) and the percentages of neutrophils, lymphocytes, and monocytes], inflammation- and coagulation-related indicators [C-reactive protein (CRP) and D-dimer], and liver function or related biochemical indicators [total protein, albumin, total bilirubin, alkaline phosphatase (ALP), gamma-glutamyl transferase (GGT), lactate dehydrogenase (LDH), and creatine kinase-MB (CK-MB)].

A complete hematological panel, including eosinophil and basophil counts or percentages, red blood cell count, hemoglobin, hematocrit, red cell indices, red cell distribution width, platelet count, and platelet-related indices, was not uniformly available as a prespecified set of candidate predictors for all participants. After data extraction, variable types and missing-data distributions were verified. Continuous variables stored as text or containing non-numeric characters were cleaned and converted to numeric format, and categorical variables were coded according to prespecified levels before statistical analysis.

Pulmonary and extrapulmonary manifestations were retrospectively identified from medical records and imaging reports for descriptive characterization of the cohort. Pulmonary manifestations included cough, fever at admission, wheezing, dyspnea, pulmonary rales, lung consolidation, and pleural effusion. Extrapulmonary manifestations included gastrointestinal symptoms, myocardial involvement, skin rash, neurological symptoms, hematological or coagulation abnormalities, and thromboembolic events. A manifestation was recorded as present when supported by clinical documentation, physical examination, laboratory findings, or imaging reports. When the available information was insufficient to confirm its presence or absence, the item was classified as missing or uncertain. These manifestations were summarized descriptively and were not included as candidate predictors in model development.

Gastrointestinal symptoms included abdominal pain, nausea, vomiting, diarrhea, or anorexia. Myocardial involvement was defined by abnormal myocardial enzyme levels and/or an explicit clinical diagnosis of myocardial injury. Hematological or coagulation abnormalities were defined as clinically documented hematological abnormalities, coagulation dysfunction, or D-dimer levels above the upper reference limit of the hospital laboratory during hospitalization.

### Laboratory measurements

Venous blood samples were collected in the early morning of the day after admission after an overnight fast, in accordance with routine clinical practice. Samples were processed and analyzed by the hospital clinical laboratory using automated biochemical and hematological analyzers and standard laboratory procedures. CRP and D-dimer were measured using the routinely established methods of the hospital laboratory. All laboratory procedures were performed by trained personnel in accordance with the manufacturers' instructions and institutional standard operating procedures. Test results were interpreted according to the local laboratory reference intervals and were verified during data extraction to reduce information bias.

### Definition of liver injury

Acute liver injury was defined primarily according to biochemical evidence of hepatocellular injury on the day of admission or the next day, namely elevated serum ALT with or without elevated AST, exceeding the upper limits of the local laboratory reference values (ALT: 40 U/L; AST: 35 U/L). Based on this definition, children were divided into a non-liver injury group and a liver injury group for subsequent model construction and validation. Although ALT and AST were used as the primary indicators for outcome definition, other liver function-related or biochemical indicators, including total protein, albumin, total bilirubin, ALP, GGT, LDH, and CK-MB, were also analyzed to capture additional aspects of hepatobiliary function, cholestatic abnormalities, hepatic synthetic status, and systemic tissue injury.

### Statistical analysis

All statistical analyses were performed using R software (R version 4.0.2; R Foundation for Statistical Computing, Vienna, Austria). First, structural verification of raw data, unification of variable types, and assessment of missing data were conducted; necessary numerical cleaning and format standardization were performed for continuous variables, and hierarchical level setting was carried out for categorical variables. A multiple imputation strategy was adopted to generate a complete dataset for missing data, and the rationality of imputation was evaluated by comparing the overall distribution characteristics of key indicators before and after imputation. Subsequently, the sample was divided into a training set and a validation set at a ratio of 7:3 by stratified random sampling based on outcomes, and baseline characteristics between the two sets were compared: continuous variables with approximately normal distribution were expressed as mean ± standard deviation and analyzed using independent samples *t*-test; non-normally distributed variables were expressed as median (interquartile range) and analyzed using Wilcoxon rank-sum test; categorical variables were expressed as number (percentage) and analyzed using chi-square test. Pulmonary and extrapulmonary manifestations were summarized as *n* (%) according to liver injury status, and the number of patients with missing or uncertain information was reported for each manifestation. Because these variables were included solely to describe the clinical characteristics of the cohort, no inferential comparisons between the liver injury and non-liver injury groups were performed. For model construction, LASSO-logistic regression was first performed in the training set for variable selection. LASSO is a penalized regression approach that introduces an L1 penalty to shrink regression coefficients, allowing less informative variables to be reduced to zero and thereby improving model parsimony when multiple candidate predictors are considered (15). The optimal penalty parameter *λ* was selected using 10-fold cross-validation. Variables with non-zero coefficients at the selected *λ* value were retained as candidate predictors and subsequently entered into a multivariable logistic regression model. A nomogram was then constructed based on the final multivariable model for individualized risk prediction. The discrimination of the model was evaluated using the receiver operating characteristic (ROC) curve, and the area under the curve (AUC) and 95% confidence interval (95% CI) of the training set and validation set were calculated; accuracy, sensitivity, and specificity were reported at the optimal cutoff value. The calibration of the model was internally calibrated by bootstrap resampling in the training set, and the consistency was evaluated based on predicted probability and actual outcomes in the validation set. The clinical application value was assessed using decision curve analysis (DCA), which calculated the net benefit at different threshold probabilities and compared it with the “intervene all/intervene none” strategies. All statistical tests were two-tailed, and a *P*-value < 0.05 was considered statistically significant.

## Results

### Study population and baseline characteristics

A total of 964 children hospitalized and diagnosed with MPP at Wuhan Third Hospital from January 2021 to December 2024 were enrolled in this study. Among them, 131 cases developed acute liver injury, with an incidence rate of 13.6% (131/964). The cohort was divided into a training set of 676 children and a validation set of 288 children. Acute liver injury occurred in 92 children (13.6%) in the training set and 39 children (13.5%) in the validation set. There were no significant differences between the two sets in gender, age, fever (body temperature ≥37.5 °C), history of allergy, or major laboratory indicators, indicating comparable baseline characteristics after random splitting (*P* > 0.05 for all, Table 1). All included children had radiological evidence of pneumonia, as required by the diagnostic criteria for MPP.

**Table 1:** Baseline characteristics of children with *Mycoplasma pneumoniae* pneumonia in the training and validation sets.

Variable	Overall	Training	Validation	*t/Z/χ* ^2^	*P*
Gender (Female)	470 (48.8)	328 (48.5)	142 (49.3)	0.023	0.879
Age (years)	6.72 (2.76)	6.69 (2.81)	6.79 (2.65)	0.529	0.606
Fever (≥37.5 °C)	125 (13.0)	90 (13.3)	35 (12.2)	0.149	0.699
Allergy History	18 (1.9)	10 (1.5)	8 (2.8)	1.217	0.270
WBC (10^9/L)	8.90 (3.82)	8.94 (3.87)	8.79 (3.72)	0.560	0.582
Neutrophils (%)	56.78 (15.48)	56.37 (15.62)	57.73 (15.16)	1.263	0.212
Lymphocytes (%)	34.27 (14.16)	34.65 (14.24)	33.36 (13.97)	1.309	0.194
Monocytes (%)	7.48 (3.02)	7.55 (3.07)	7.32 (2.91)	1.108	0.278
D-dimer (mg/L)	0.32 [0.23, 0.46]	0.33 [0.23, 0.46]	0.32 [0.23, 0.45]	0.777	0.437
CRP (mg/L)	1.52 [0.29, 6.81]	1.56 [0.28, 6.20]	1.48 [0.30, 8.33]	0.752	0.451
Total protein (g/L)	69.34 (4.50)	69.38 (4.45)	69.26 (4.63)	0.384	0.697
Albumin (g/L)	42.60 (2.73)	42.68 (2.70)	42.40 (2.78)	1.490	0.132
Total bilirubin (μmol/L)	5.50 [4.20, 7.40]	5.40 [4.18, 7.40]	5.60 [4.30, 7.30]	0.840	0.401
ALP (U/L)	172.05 (49.33)	173.61 (49.87)	168.41 (47.94)	1.521	0.135
GGT (U/L)	10.00 [8.00, 12.00]	10.00 [8.00, 12.00]	10.00 [8.00, 12.00]	0.300	0.762
LDH (U/L)	262.00 [218.00, 318.00]	264.00 [218.00, 318.00]	259.50 [218.00, 320.00]	0.244	0.807
CK-MB (U/L)	22.00 [15.00, 33.00]	22.00 [15.00, 33.00]	22.00 [14.75, 32.25]	0.000	1.000

Data are presented as mean ± standard deviation (SD), median with interquartile range (IQR), or *n* (%), as appropriate. Normally distributed continuous variables were compared using the independent-samples t test, non-normally distributed continuous variables were compared using the Wilcoxon rank-sum test, and categorical variables were compared using the chi-square test or Fisher's exact test, as appropriate. WBC, white blood cell count; CRP, C-reactive protein; ALP, alkaline phosphatase; GGT, gamma-glutamyl transferase; LDH, lactate dehydrogenase; CK-MB, creatine kinase-MB.

### Clinical manifestations of the study cohort

The pulmonary and extrapulmonary manifestations documented in the available records are summarized in Supplementary Table S1. In addition to acute liver injury, the recorded extrapulmonary manifestations included gastrointestinal symptoms, hematological or coagulation abnormalities, myocardial involvement, skin rash, neurological symptoms, and thromboembolic events. At least one extrapulmonary manifestation other than liver injury was documented in 132 children (13.7%), including 88 of 833 children (10.6%) without liver injury and 44 of 131 children (33.6%) with liver injury. Two or more extrapulmonary manifestations were documented in 71 children (7.4%), including 45 children (5.4%) without liver injury and 26 children (19.8%) with liver injury. Supplementary Table S1 also reports the number of patients with missing or uncertain information for each manifestation.

### Variable selection and model development

**Figure 1:**
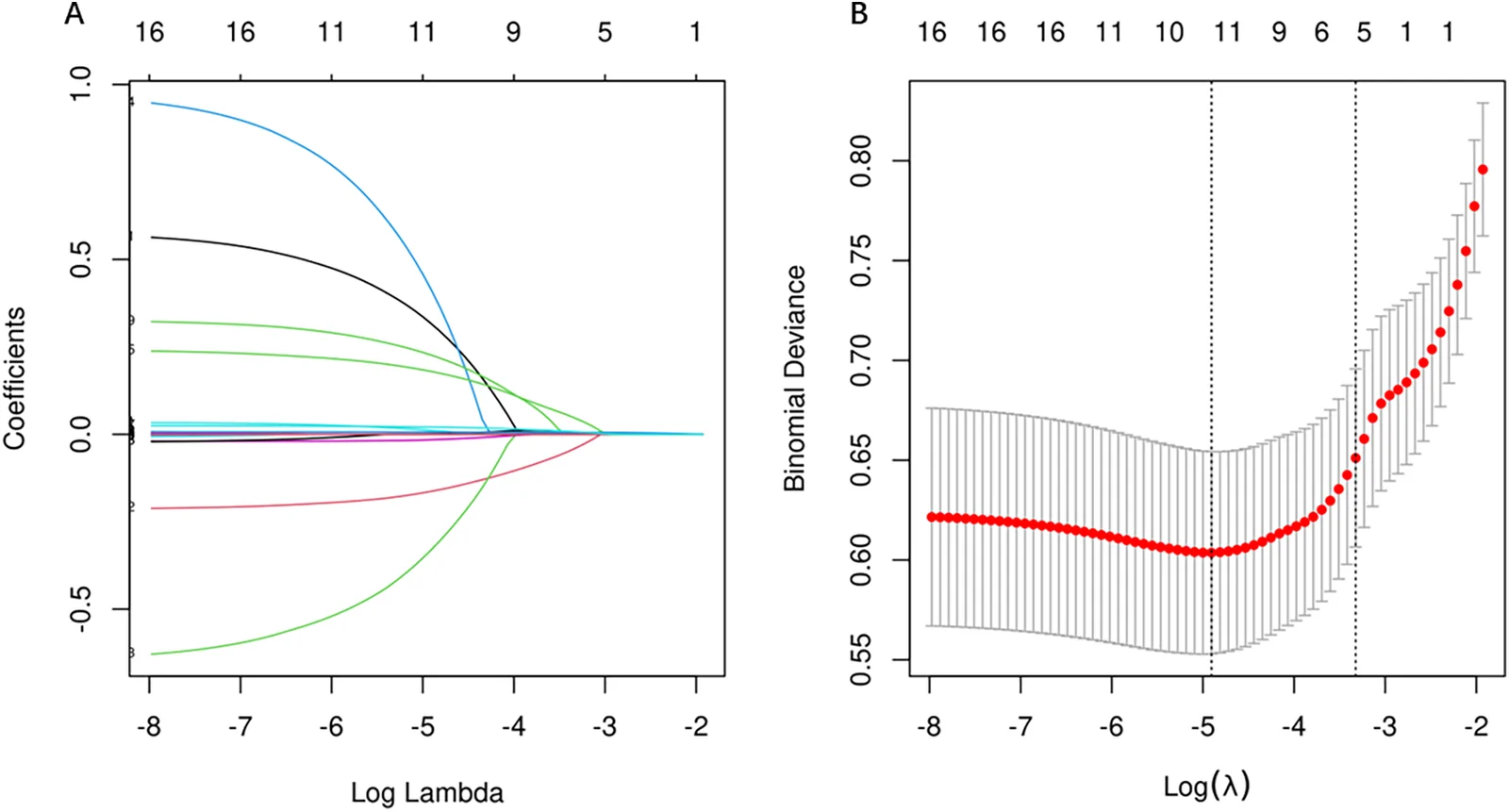
LASSO regression for feature selection in the training set. **(A)** Coefficient profiles of candidate predictors against log(*λ*). **(B)** Ten-fold cross-validation curve for selecting the optimal penalty parameter *λ*; λ_min was used to determine predictors with non-zero coefficients.

Univariable logistic regression analysis was first performed in the training set. The results showed that age, neutrophil percentage, lymphocyte percentage, D-dimer, total protein, *γ*-glutamyl transferase, lactate dehydrogenase, and CK-MB were associated with the occurrence of liver injury (*P* < 0.05 for all, Table 2). Subsequently, LASSO regression was used to screen candidate variables, and the optimal penalty parameter was determined by 10-fold cross-validation. Eleven predictors with non-zero coefficients were obtained in the model corresponding to lambda.min (Figure 1), including gender, age, body temperature ≥37.5 °C, history of allergy, neutrophil percentage, lymphocyte percentage, D-dimer, total protein, *γ*-glutamyl transferase, lactate dehydrogenase, and CK-MB.

**Table 2:** Univariable logistic regression analysis of factors associated with acute liver injury in children with *Mycoplasma pneumoniae* pneumonia (training set).

Variable	Beta	SE	OR (95% CI)	*P*
Gender (Female)	0.270	0.225	1.31 (0.843–2.037)	0.230
Age (years)	−0.201	0.042	0.818 (0.753–0.889)	<0.001
Fever (≥37.5 °C)	−0.395	0.371	0.673 (0.326–1.393)	0.286
Allergy History (Yes)	1.022	0.699	2.778 (0.705–10.943)	0.144
WBC	0.034	0.028	1.035 (0.979–1.093)	0.225
Neutrophil (%)	−0.025	0.007	0.975 (0.962–0.989)	<0.001
Lymphocyte (%)	0.028	0.008	1.028 (1.013–1.044)	<0.001
Monocyte (%)	0.009	0.036	1.009 (0.94–1.084)	0.797
D-dimer (mg/L)	0.417	0.183	1.518 (1.059–2.175)	0.023
CRP (mg/L)	0.000	0.012	1 (0.977–1.024)	0.973
Total Protein (g/L)	0.061	0.025	1.063 (1.012–1.116)	0.015
Albumin (g/L)	0.040	0.041	1.041 (0.96–1.129)	0.333
Total Bilirubin (μmol/L)	−0.051	0.040	0.951 (0.878–1.029)	0.211
ALP (U/L)	−0.001	0.002	0.999 (0.995–1.004)	0.714
GGT (U/L)	0.061	0.028	1.063 (1.007–1.122)	0.027
LDH (U/L)	0.010	0.001	1.01 (1.007–1.012)	<0.001
CK-MB (U/L)	0.034	0.005	1.035 (1.025–1.044)	<0.001

OR, odds ratio; CI, confidence interval. ORs are reported per 1-unit increase for continuous variables and relative to the reference category for categorical variables.

### Multivariable logistic regression and nomogram construction

**Figure 2:**
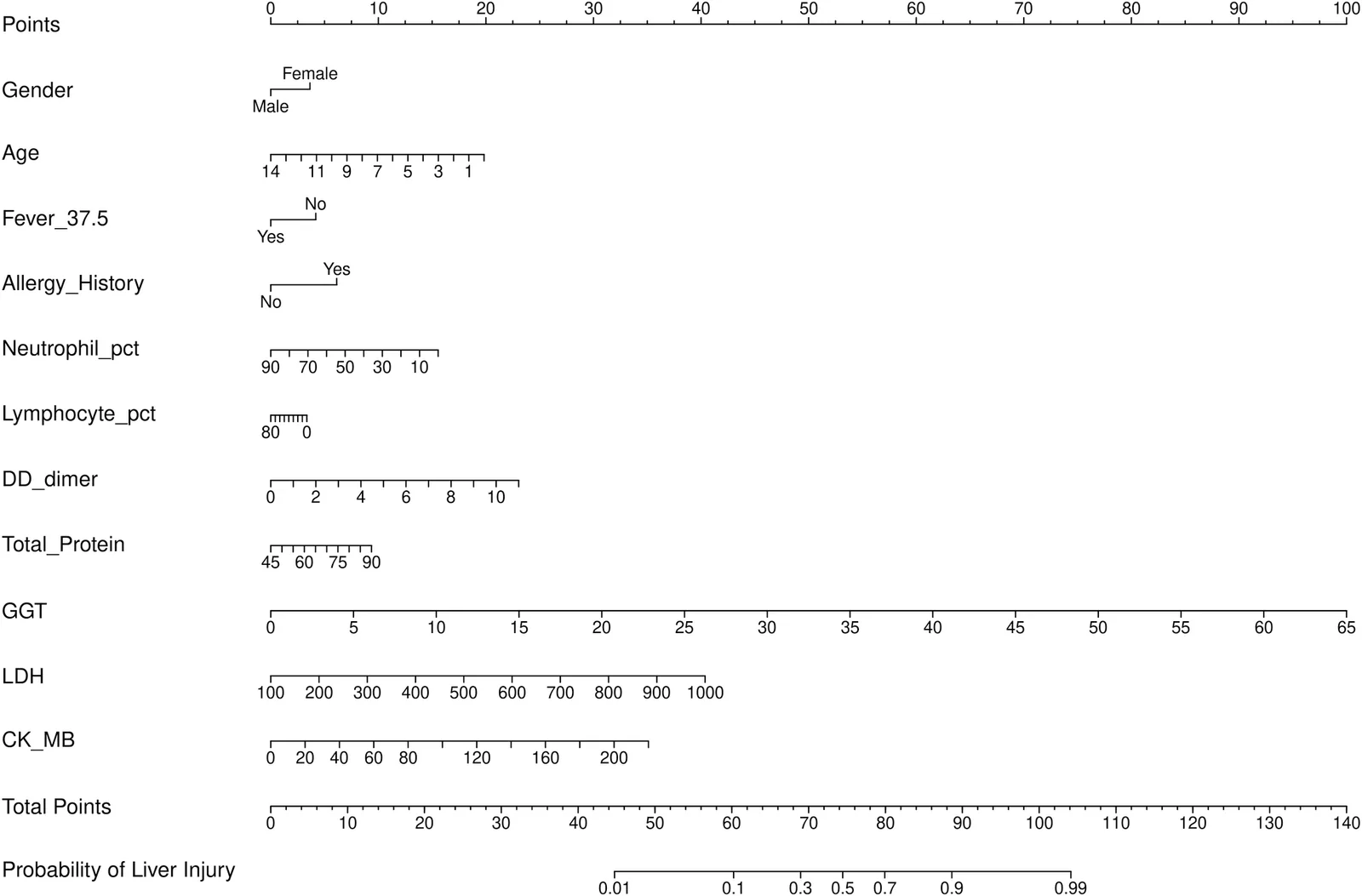
Nomogram for predicting acute liver injury in children with *Mycoplasma pneumoniae* pneumonia. For each predictor, draw a vertical line to the “Points” axis to obtain points; sum all points to obtain “Total Points,” and project to the probability scale to estimate the individual risk.

The above 11 variables were included in multivariate logistic regression to establish the prediction model. The results indicated that female gender (OR = 1.757, 95% CI: 1.013–3.046, *P* = 0.045), *γ*-glutamyl transferase (OR = 1.269, 95% CI: 1.158–1.390, *P* < 0.001), lactate dehydrogenase (OR = 1.007, 95% CI: 1.004–1.010, *P* < 0.001), and CK-MB (OR = 1.025, 95% CI: 1.012–1.038, *P* < 0.001) were independently associated with an increased risk of liver injury, while age was negatively associated with liver injury (OR = 0.803, 95% CI: 0.717–0.900, *P* < 0.001). D-dimer showed a marginal association in the multivariable model but did not reach conventional statistical significance (OR = 1.383, 95% CI: 0.995–1.922, *P* = 0.053) (Table 3). A nomogram was constructed based on the multivariate model, in which the total score was obtained by summing the scores corresponding to each predictive variable, and the predicted probability of individual liver injury was calculated accordingly (Figure 2).

**Table 3:** Multivariable logistic regression model for predicting acute liver injury in children with *Mycoplasma pneumoniae* pneumonia (training set).

Variable	Beta	SE	OR (95% CI)	*P*
Gender (Female)	0.564	0.281	1.757 (1.013–3.046)	0.045
Age (years)	−0.219	0.058	0.803 (0.717–0.9)	<0.001
Fever (≥37.5 °C)	−0.647	0.480	0.523 (0.204–1.34)	0.177
Allergy History (Yes)	0.949	0.931	2.582 (0.417–16.001)	0.308
Neutrophil (%)	−0.027	0.037	0.974 (0.905–1.047)	0.470
Lymphocyte (%)	−0.006	0.040	0.994 (0.919–1.074)	0.871
D-dimer (mg/L)	0.324	0.168	1.383 (0.995–1.922)	0.053
Total Protein (g/L)	0.032	0.034	1.033 (0.966–1.104)	0.343
GGT (U/L)	0.238	0.047	1.269 (1.158–1.39)	<0.001
LDH (U/L)	0.007	0.002	1.007 (1.004–1.01)	<0.001
CK-MB (U/L)	0.025	0.007	1.025 (1.012–1.038)	<0.001

OR, odds ratio; CI, confidence interval. Variables included were selected by LASSO regression and entered into the multivariable logistic regression model.

### Model performance and internal validation

**Figure 3:**
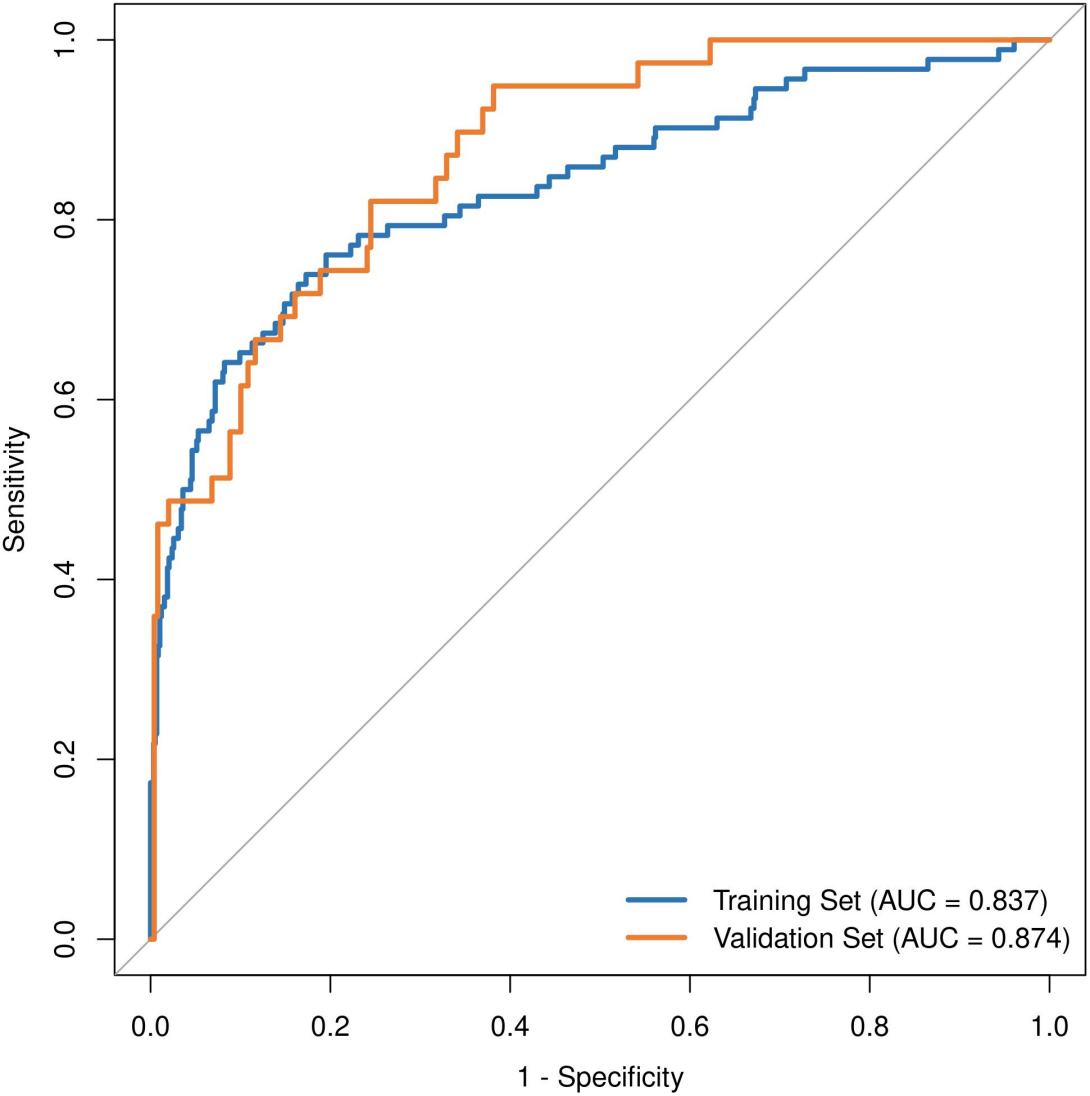
Receiver operating characteristic (ROC) curves of the nomogram in the training and validation sets.

**Figure 4:**
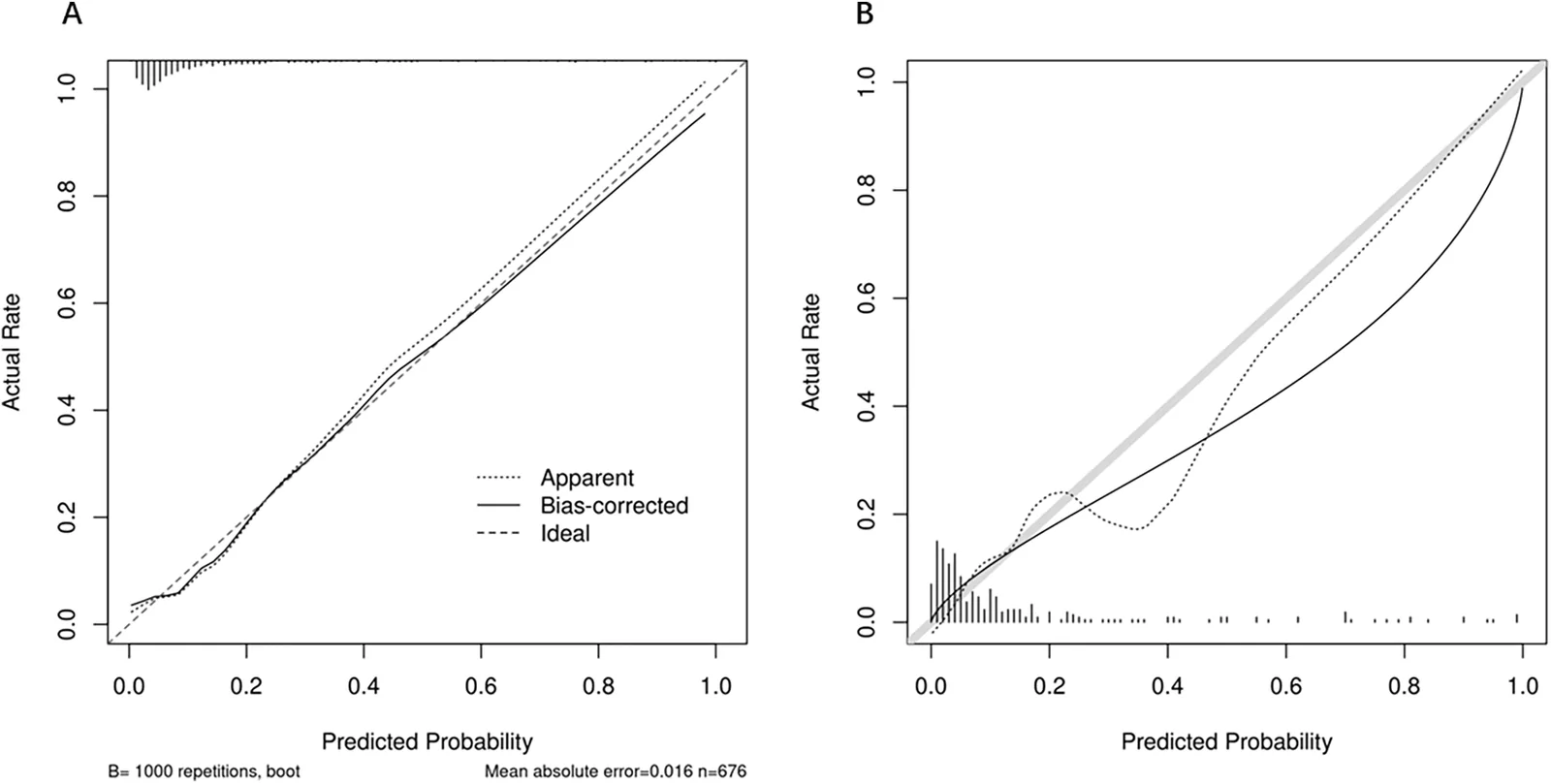
Calibration curves of the nomogram. **(A)** Training set (bootstrap-corrected calibration). **(B)** Validation set calibration comparing predicted probabilities with observed outcomes; the 45° line represents perfect calibration.

**Figure 5:**
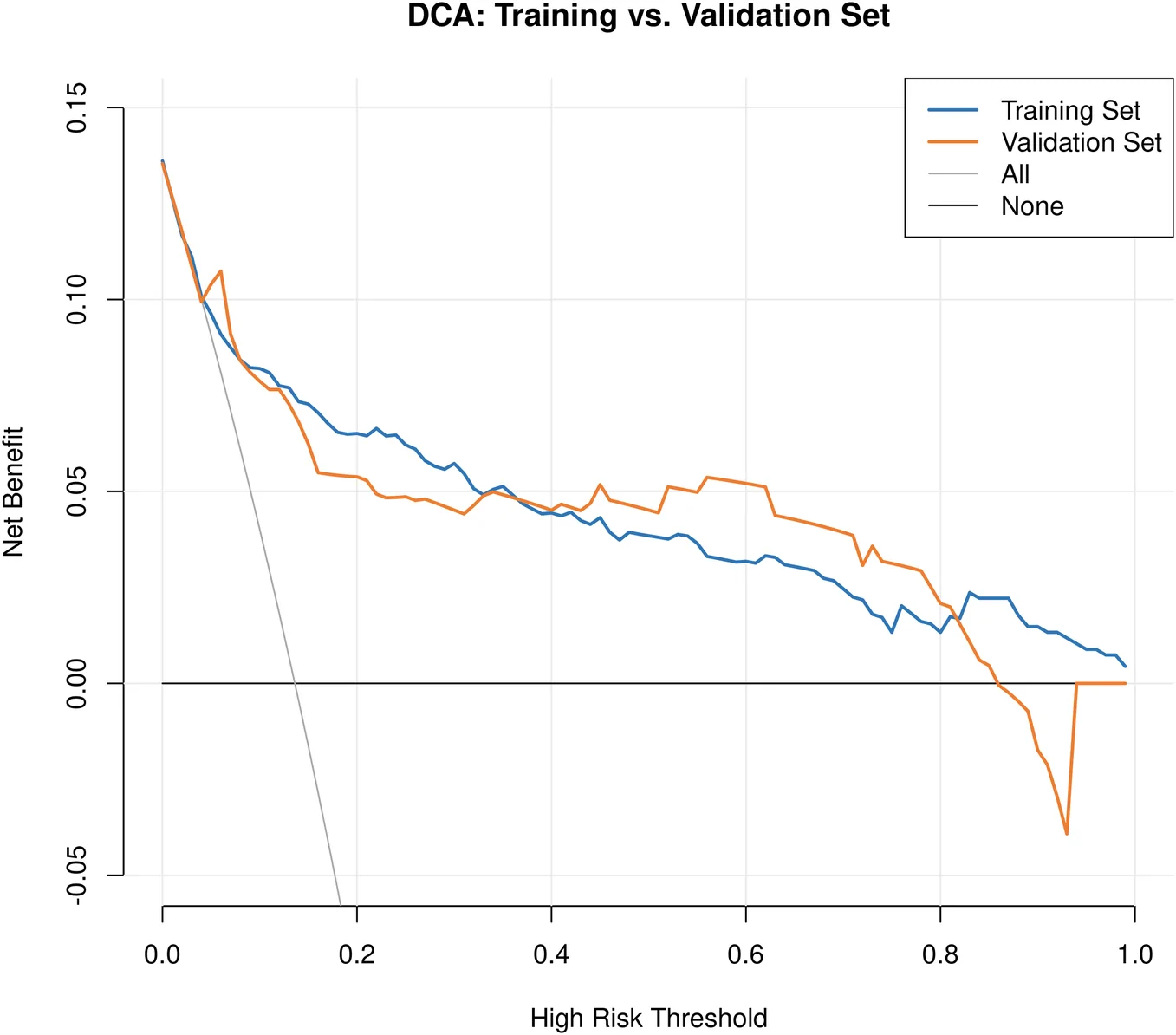
Decision curve analysis (DCA) of the nomogram in the training and validation sets. Net benefit across threshold probabilities is shown for the model and reference strategies (treat-all and treat-none).

The model discrimination assessment showed that the AUC was 0.837 (95% CI: 0.786–0.889) in the training set and 0.874 (95% CI: 0.819–0.929) in the validation set (Figure 3). At the optimal cutoff value, the accuracy, sensitivity, and specificity were 0.815, 0.739, and 0.827 in the training set, and 0.764, 0.821, and 0.755 in the validation set, respectively (Table 4), suggesting that the model had good discriminative ability in both training and validation sets. The calibration curves showed that the bootstrap-corrected predicted probability was generally consistent with the actual incidence in the training set, and the validation set also showed good fitting consistency (Figure 4). Decision curve analysis demonstrated that within a certain range of threshold probabilities, the model achieved higher net benefit than the “treat all” or “treat none” strategies, indicating the potential clinical application value of this nomogram (Figure 5).

**Table 4:** Predictive performance of the nomogram in the training and validation sets.

Metric	Training Set	Validation Set
AUC (95% CI)	0.837 (0.786–0.889)	0.874 (0.819–0.929)
Accuracy	0.815	0.764
Sensitivity	0.739	0.821
Specificity	0.827	0.755

AUC, area under the receiver operating characteristic curve; CI, confidence interval. Accuracy, sensitivity, and specificity were calculated at the optimal cutoff determined by the Youden index.

## Discussion

This study was based on a cohort of hospitalized children with MPP, and a nomogram was constructed using LASSO regression combined with multivariate Logistic regression to predict acute liver injury in MPP. The results showed that the model exhibited good discrimination in both the training and validation sets (AUC approximately 0.84–0.87). The calibration curves indicated favorable consistency between predicted and actual incidence rates, and DCA also showed net benefit within a certain threshold range, suggesting that this tool has clinically applicable value for risk stratification.

The differences between the univariable and multivariable logistic regression results should be interpreted in the context of adjustment for correlated predictors. Univariable logistic regression reflects the crude association between each individual variable and liver injury, whereas multivariable logistic regression estimates the independent association after simultaneous adjustment for other variables in the model. Therefore, variables that were significant in the univariable analysis, such as neutrophil percentage, lymphocyte percentage, and total protein, may no longer remain significant after adjustment because part of their association with liver injury may be explained by other related predictors. For example, neutrophil and lymphocyte percentages both reflect systemic inflammatory status and may share overlapping information with markers of disease severity or tissue injury. Similarly, total protein may be influenced by nutritional status, inflammatory burden, and hepatic synthetic function, and its crude association may be attenuated after adjustment for liver- or tissue injury-related indicators such as GGT, LDH, and CK-MB. Thus, the discrepancy between univariable and multivariable results does not indicate inconsistency, but rather reflects the distinction between crude associations and adjusted independent associations.

The key predictors retained in the multivariable model may represent different but interrelated dimensions of disease biology in children with MPP. GGT is a hepatobiliary enzyme and may reflect cholestatic involvement or hepatobiliary stress, whereas LDH is a nonspecific marker of tissue injury, inflammatory burden, and hypoxic stress (6, 7, 10, 14). CK-MB may indicate concomitant myocardial involvement or systemic organ stress, which is consistent with previous evidence that myocardial injury can occur as an extrapulmonary manifestation of MPP (16–18). D-dimer, although only marginally associated with liver injury in the present multivariable model, may reflect activation of the inflammation-coagulation axis, endothelial injury, or microcirculatory disturbance in more severe MPP phenotypes (3, 19). The inverse association between age and liver injury observed in this cohort suggests that age-related differences may contribute to risk stratification; however, the underlying biological mechanisms could not be determined from the present retrospective data and require further investigation. Taken together, these variables may jointly characterize a systemic inflammatory, coagulation-activated, and multi-organ stress phenotype rather than acting as isolated or fully independent pathological processes.

According to previous evidence, the core characteristics of extrapulmonary involvement in MPP include systemic inflammatory activation and immune imbalance, which can further trigger coagulation, endothelial, and multi-organ stress responses. In this study, neutrophil and lymphocyte percentages were included in the model, consistent with the relevance of inflammatory profiles reported in previous studies on severe/refractory MPP. Related studies have shown that refractory MPP is characterized by marked immune activation accompanied by pathological processes such as mucus plug formation. The immune activation and abnormal mucin secretion reported by Liu et al. (20) provide mechanistic clues for “inflammation-driven extrapulmonary injury”. Meanwhile, in populations with coinfection or more complex pathogen profiles, routine blood and biochemical indicators fluctuate more significantly, suggesting a concurrent increase in inflammatory burden and organ stress (21). Furthermore, Liu et al. (22) proposed a possible threshold effect of white blood cell count on the risk of refractory MPP, which may also explain why the strength of association between inflammatory indicators and complications varies across studies: once inflammation exceeds a critical threshold, the risk of extrapulmonary organ involvement may increase nonlinearly.

Coagulation activation represents another important signal in this study. The inclusion of D-dimer in the model suggests that the inflammation-coagulation axis may be associated with the development of liver injury. Previous case series and reviews have shown that MPP can be complicated by thrombotic events such as pulmonary embolism, highlighting the clinical significance of endothelial injury and hypercoagulability in pediatric MPP (3, 5). More specifically, Yue et al. (19) demonstrated that D-dimer can predict severe phenotypes such as plastic bronchitis or necrotizing pneumonia, indicating its role as a surrogate marker for severe biological phenotypes. The liver is sensitive to microcirculatory disturbance and inflammation-mediated injury; thus, the association between D-dimer and liver injury risk is biologically plausible.

For hepatobiliary-related indicators, GGT and total protein were included in the model, suggesting that cholestasis, hepatic synthetic function, and systemic inflammatory stress may jointly contribute to the liver injury phenotype. Previous studies have reported both MP-related hepatitis and cases presenting with hepatitis or cholestasis without obvious pulmonary involvement (23, 24), supporting that MP can affect the hepatobiliary system through immune-mediated mechanisms, molecular mimicry, or indirect inflammatory injury. On the other hand, cases of Kawasaki disease with cholestatic hepatitis and concurrent MP infection suggest that some children may have a stronger immune-inflammatory background, leading to more complex hepatobiliary involvement (25). These discrepancies imply that variations in the incidence and severity of liver injury across studies may be related to differences in the enrolled population (proportion of severe cases), definition of liver injury (hepatocellular injury vs. cholestatic type), and timing of detection, which require further standardization in future research.

The inclusion of LDH and CK-MB suggests that tissue injury and multi-organ involvement may represent important accompanying features of liver injury risk. LDH reflects generalized tissue damage and hypoxic stress, and has been proven to have predictive value in studies of infection-related liver injury. For example, Xu et al. (14) reported that the combination of LDH and metabolic-related indicators improved predictive performance in pediatric sepsis-associated liver injury. CK-MB is associated with myocardial injury, and myocardial involvement in MPP has been documented in multiple studies. Combined detection of N-terminal pro-brain natriuretic peptide (NT-proBNP) and cardiac troponin I (cTnI) shows good efficacy in diagnosing myocardial injury (16), and several studies have attempted to construct risk prediction models for myocardial injury (17) or explore the correlation between inflammatory factors and myocardial injury (18). Therefore, the inclusion of CK-MB in the liver injury model more likely represents a severe phenotype characterized by “resonance between systemic inflammation and organ injury” rather than a unidirectional causal chain.

Compared with previous modeling studies, the clinical advantages of the present study lie in interpretability and clinical feasibility. Previous studies have attempted to use machine learning to establish models for differential diagnosis of severe MPP or prediction of organ injury risk (13), and others have used CT radiomics to assess the risk of extrapulmonary organ involvement (26). Although these methods have potential for performance improvement, they often rely on more complex data and procedures, with limited clinical interpretability and reproducibility. In contrast, the present study constructed a nomogram using routine clinical and laboratory indicators, facilitating rapid bedside calculation and communication, and is suitable for early identification of high-risk children and guidance of monitoring strategies. Notably, associations between radiological severe phenotypes (such as lung consolidation, necrotizing pneumonia) and complication risk have been reported (27). The lack of systematic inclusion of radiological quantitative features in this study may have sacrificed some information gain. Future studies may explore integrated modeling combining radiological information and laboratory indicators to achieve more comprehensive risk characterization.

This study has several limitations. First, the retrospective design inevitably introduced selection bias and information bias, and confounding factors such as medication exposure, previous liver disease, and nutritional status may have been incompletely recorded. Although the pulmonary and extrapulmonary manifestations identifiable from the available records were summarized in Supplementary Table S1, these manifestations were not collected prospectively using standardized definitions or a uniform case-report form. The completeness of documentation may therefore have differed among patients and across organ systems, resulting in possible under-ascertainment or misclassification. In particular, absence of documentation could not always be interpreted as confirmed absence of the corresponding manifestation. Accordingly, the frequencies reported in Supplementary Table S1 should be regarded as a descriptive overview of documented manifestations rather than as complete incidence estimates. Second, the definition and timing of liver injury detection may affect outcome classification, leading to limited cross-study comparability, especially regarding phenotypic differences between cholestasis and hepatocellular injury (24, 25).

Third, several variables included in the present model, including neutrophil and lymphocyte percentages, D-dimer, and CK-MB, may vary during the clinical course of MPP. Serial changes in inflammatory and coagulation-related markers may reflect evolving disease activity, endothelial or coagulation activation, and organ stress more comprehensively than a single admission measurement. Previous studies have associated elevated D-dimer levels with severe MPP, more pronounced clinical manifestations, and pulmonary or extrapulmonary complications, whereas CK-MB is primarily interpreted as a marker of concomitant myocardial injury or systemic organ stress (16–19). Therefore, reliance on a single admission value may have resulted in incomplete characterization of the dynamic risk profile.

In addition, a complete hematological panel, including eosinophil and basophil counts or percentages, red blood cell count, hemoglobin, hematocrit, red cell indices, red cell distribution width, platelet count, and platelet-related indices, was not uniformly extracted as a prespecified set of candidate predictors. These variables may provide complementary information regarding atopy-related immune responses, anemia or altered oxygen-carrying capacity, systemic inflammation, platelet activation, and coagulation status. Their absence prevented us from evaluating whether they provided incremental predictive information beyond the routinely available blood cell parameters included in the present model.

Serum immunoglobulins and total IgE were also not systematically measured. Previous studies have reported higher total serum IgE levels in children with *Mycoplasma pneumoniae*-related extrapulmonary diseases than in those with respiratory manifestations alone, and have suggested that atopy may be associated with greater disease severity and extrapulmonary involvement (28–30). Humoral immune indices, including IgA and IgM, have also been reported to vary with MPP severity and to be associated with liver biochemical abnormalities (31). Consequently, the absence of these immunological variables limited our ability to characterize host immune susceptibility and to determine whether they provide additional predictive information beyond routinely available clinical and laboratory indicators. Prospective studies incorporating serial hematological, coagulation, cardiac, and immunological measurements are warranted to evaluate their incremental value and improve the biological interpretation of the model.

Fourth, larger-sample, multicenter, prospective external validation is required to assess generalizability and perform head-to-head comparisons with radiomics or machine learning models (13, 26). Finally, the risk spectrum may differ in subgroups with special immune activation status (such as infection-associated hemophagocytic syndrome) complicated by liver injury. Related studies have suggested that ferritin and other indicators are associated with severe infection-related immune activation and liver injury, indicating that subgroup recalibration or stratified model optimization may be conducted in the future (32).

## Conclusion

This study developed an internally validated nomogram for predicting acute liver injury in children with MPP using routinely available clinical and laboratory variables. The model showed favorable discrimination, calibration, and potential clinical net benefit, suggesting that it may help identify children at increased risk and guide early liver function monitoring. Further prospective, multicenter external validation is needed before broad clinical application.

## Data Availability

The original contributions presented in the study are included in the article/Supplementary Material, further inquiries can be directed to the corresponding author.
