# Supplementary material for: Development of a nomogram to predict acute liver injury in children with Mycoplasma pneumoniae pneumonia
Source: Front Pediatr. 2026 Aug 18;14:1809544. doi: 10.3389/fped.2026.1809544 (PMC13529755; doi:10.3389/fped.2026.1809544)
Supplement: Supplementary file 1 [file Table1.docx]

**Supplementary Table S1. Documented pulmonary and extrapulmonary manifestations in children with *Mycoplasma pneumoniae pneumonia* according to liver injury status**

| **Clinical manifestation** | **Overall (n = 964)** | **Without liver injury (n = 833)** | **With liver injury (n = 131)** | **Missing/uncertain information, n** |
| --- | --- | --- | --- | --- |
| **Pulmonary manifestations** | | | | |
| Cough | 891 (92.4) | 766 (92.0) | 125 (95.4) | 7 |
| Body temperature >= 37.5 °C at admission | 125 (13.0) | 103 (12.4) | 22 (16.8) | 0 |
| Wheezing | 164 (17.0) | 137 (16.4) | 27 (20.6) | 18 |
| Shortness of breath or dyspnea | 77 (8.0) | 60 (7.2) | 17 (13.0) | 14 |
| Pulmonary rales | 533 (55.3) | 455 (54.6) | 78 (59.5) | 9 |
| Radiological evidence of pneumonia | 964 (100.0) | 833 (100.0) | 131 (100.0) | 0 |
| Lung consolidation | 268 (27.8) | 218 (26.2) | 50 (38.2) | 0 |
| Pleural effusion | 62 (6.4) | 47 (5.6) | 15 (11.5) | 0 |
| **Extrapulmonary manifestations** | | | | |
| Acute liver injury | 131 (13.6) | 0 (0.0) | 131 (100.0) | 0 |
| Gastrointestinal symptoms | 155 (16.1) | 119 (14.3) | 36 (27.5) | 21 |
| Myocardial involvement | 66 (6.8) | 45 (5.4) | 21 (16.0) | 26 |
| Skin rash | 38 (3.9) | 31 (3.7) | 7 (5.3) | 12 |
| Neurological symptoms | 14 (1.5) | 10 (1.2) | 4 (3.1) | 17 |
| Hematological/coagulation abnormalities | 72 (7.5) | 49 (5.9) | 23 (17.6) | 24 |
| Thromboembolic events | 5 (0.5) | 3 (0.4) | 2 (1.5) | 4 |
| Any extrapulmonary manifestation other than liver injury | 132 (13.7) | 88 (10.6) | 44 (33.6) | 32 |
| Two or more extrapulmonary manifestations | 71 (7.4) | 45 (5.4) | 26 (19.8) | 35 |

Footnote: Data are presented as n (%). Pulmonary and extrapulmonary manifestations were retrospectively extracted from medical records and imaging reports. The reported frequencies represent manifestations explicitly documented in the available records and should not be interpreted as complete incidence estimates. Individual patients could have more than one manifestation; therefore, the sum of individual categories may exceed the total number of patients. “Missing/uncertain information” indicates that the available records were insufficient to confirm either the presence or absence of the corresponding manifestation. Radiological evidence of pneumonia was present in all included patients because it was part of the diagnostic criteria for Mycoplasma pneumoniae pneumonia. Gastrointestinal symptoms included abdominal pain, nausea, vomiting, diarrhea, or anorexia. Myocardial involvement was defined based on abnormal myocardial enzyme markers and/or clinical documentation of myocardial injury. Hematological/coagulation abnormalities included clinically documented coagulation abnormalities, markedly elevated D-dimer, or hematological abnormalities recorded during hospitalization. “Any extrapulmonary manifestation other than liver injury” and “two or more extrapulmonary manifestations” were used to describe the coexistence of extrapulmonary involvement.
